# Knowledge translation tool to improve pregnant women’s awareness of gestational weight gain goals and risks of gaining outside recommendations: a non-randomized intervention study

**DOI:** 10.1186/s12884-015-0534-z

**Published:** 2015-04-30

**Authors:** Sarah D McDonald, Christina K Park, Eleanor Pullenayegum, Keyna Bracken, Wendy Sword, Helen McDonald, Binod Neupane, Valerie H Taylor, Joseph Beyene, Valerie Mueller, Melissa Brouwers

**Affiliations:** Department of Obstetrics & Gynecology, McMaster University, 1280 Main St. West, Hamilton, ON L8S 4L8 Canada; Department of Radiology, McMaster University, 1280 Main St. West, Hamilton, ON L8S 4L8 Canada; Department of Clinical Epidemiology & Biostatistics, McMaster University, 1280 Main St. West, Hamilton, ON L8S 4L8 Canada; Child Health Evaluative Sciences Program, Hospital for Sick Children, 555 University Avenue, Toronto, ON M5G 1X8 Canada; Department of Family Medicine, McMaster University, 1280 Main St. West, Hamilton, ON L8S 4L8 Canada; School of Nursing, McMaster University, Hamilton, ON L8S 4L8 Canada; Midwifery Education Program, McMaster University, 1280 Main St. West, Hamilton, ON L8S 4L8 Canada; Department of Psychiatry, Women’s College Hospital, 76 Grenville St, Toronto, ON M5S 1B1 Canada; Department of Oncology, McMaster University, 1280 Main St. West, Hamilton, ON L8S 4L8 Canada

**Keywords:** Knowledge translation, Intervention studies, Surveys, Gestational weight gain, Lifestyle factors, Counseling

## Abstract

**Background:**

There is an urgent need to prevent excessive pregnancy weight gain, a contributor to both maternal and child obesity. However, the majority of women had reported not being counseled to gain an appropriate amount of gestational weight by their health care providers. We developed a knowledge translation (KT) tool designed to facilitate the clinical interaction between pregnant women and their health care providers (HCPs). We piloted the tool on the impact on women’s knowledge of gestational weight gain (GWG) goals, and evaluated its potential in promoting appropriate knowledge about GWG within the 2009 Institute of Medicine guidelines.

**Methods:**

We conducted a prospective cohort study, comparing women’s knowledge about GWG after the KT tool to women from the same clinics and care providers the year prior. Our primary outcome was the proportion of women who reported receiving an appropriate GWG recommendation from their care provider. We evaluated knowledge on a survey conducted at enrollment in the cohort at ≤ 20 weeks gestation and evaluated participant satisfaction with the KT tool in the third trimester. We performed univariate and multivariable logistic regression analyses for differences in outcomes with historical controls from the same clinics. Our *a priori* sample size calculation required 130 participants to demonstrate a 15% increase in reported counseling about gestational weight gain.

**Results:**

One hundred and forty-six women were recruited and 131 (90%) completed the enrollment survey. Women who received the KT tool were more likely to report receiving a specific GWG recommendation from their HCP (adjusted odds ratio [AOR] 3.45, 95% confidence interval [CI] 2.22-5.37) and discussing GWG topics with their HCP (AOR 7.96, 95% CI 4.41-14.37), and believing that there were risks to their infants with inadequate GWG (AOR 2.48, 95% CI 1.14-5.37). Half of women (49.5%) indicated that they would recommend the tool to a friend.

**Conclusions:**

Women who received the KT tool reported receiving more counseling on GWG from their HCPs and were more aware of the risks of gaining outside appropriate GWG recommendations. The association between GWG education and GWG requires further research.

## Background

High weight gain during pregnancy has serious implications, including increased risk of later obesity for both mothers [1] and their infants [2]. Other risks of high gestational weight gain (GWG) include being large for gestational age [3] and preterm birth [4] for infants, and postpartum weight retention [5] and cesarean delivery [6] for mothers, which were some of the outcomes considered in the development of the 2009 Institute of Medicine (IOM) gestational weight gain guidelines, along with childhood obesity and small for gestational age [7]. Approximately 10.9% (95% CI 10.7-11.1) of cesarean sections overall were attributable to above recommended GWG and this proportion was even more striking in multiparous women with no prior cesarean at 23.6% (95% CI 23.0-24.2%) [8].

Previous work has shown that up to 88% of women reported not being counseled to gain the appropriate amount of weight by their antenatal care providers [9]. Furthermore, 70% of women indicated that either a chart or a website showing how much weight should be gained each week and in total would be helpful [9]. Over three quarters of practitioners reported that a tool that would calculate GWG would be helpful [10].

Computer- and web-based platforms are effective at influencing patient and practitioner decision behavior [11-14]. For instance, a systematic review found that computerized clinical decision support systems improved practitioner performance, leading to improved diagnosis, preventive care, disease management, drug dosing, or drug prescribing [14]. A systematic review of interactive health communication applications reported a significant positive effect on patients’ knowledge, social support, self-efficacy, and behavioral outcomes over time [13]. Another systematic review demonstrated that most patients welcomed a patient-held record [12]. As these aspects were relatively easy to implement and were sustainable, we developed a knowledge translation (KT) tool for GWG. Our tool was designed to present information in “clear, concise, and user friendly formats and ideally to provide explicit recommendations” to meet participants’ knowledge needs and influence their behavior [15].

The primary objective of this study was to pilot a KT tool designed to improve women’s knowledge of GWG, thereby assessing suitability for further study. We additionally sought to assess participants’ evaluations of the tool.

## Methods

### Study design

We conducted a prospective cohort study, comparing women receiving the KT tool (“KT group”) to historical controls (“control group”) in a non-randomized comparison. Historical controls were used as data from the same clinics from only one year prior to the present study were available to determine if the KT tool improved outcomes in women in the same geographical setting. In addition, the use of historical controls reduced the additional burden on the participating clinics and avoided contamination of the KT group into the control group.

### The intervention

**Figure 1:**
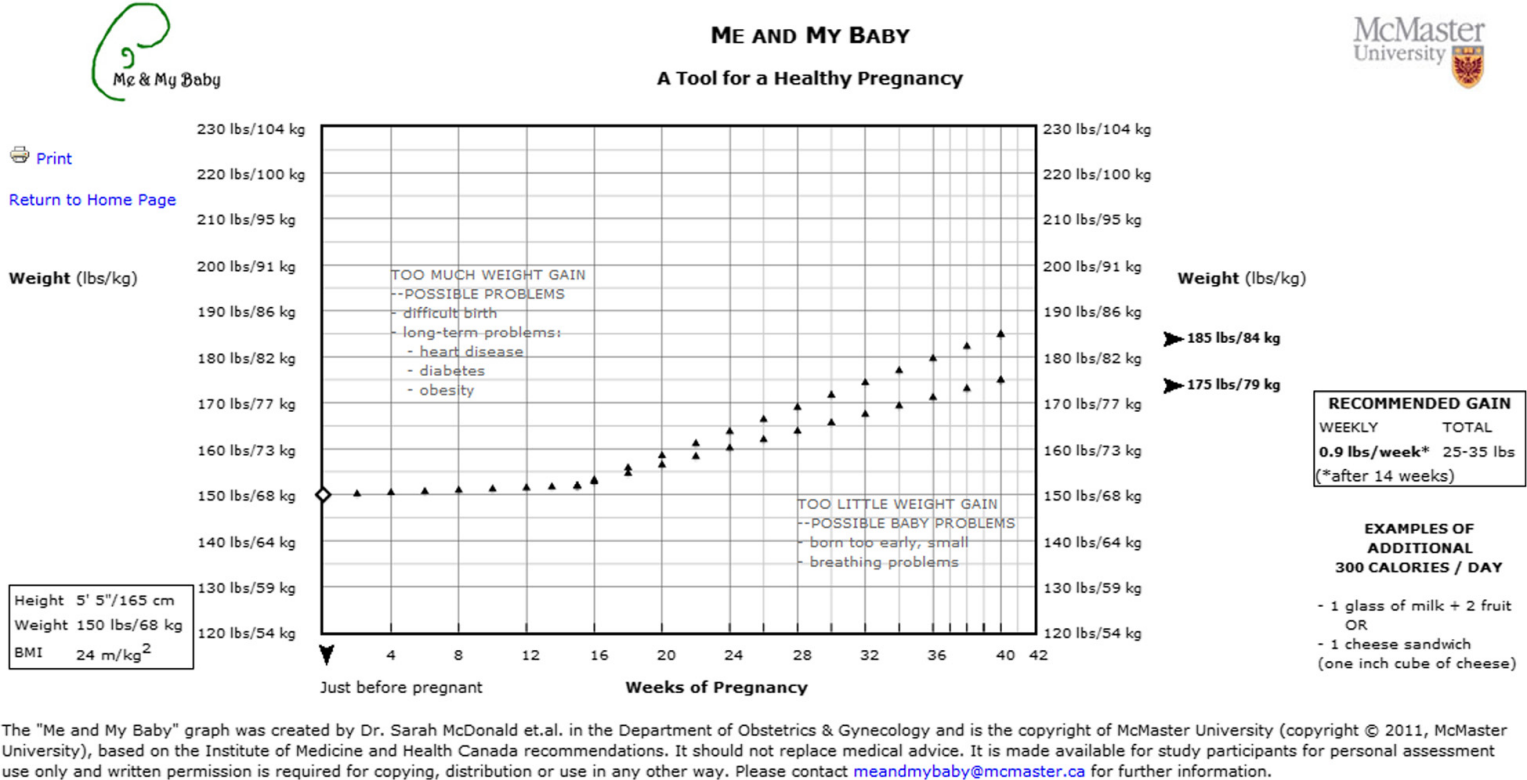
Sample of Me And My Baby knowledge translation tool. Legend: A sample Me and My Baby knowledge translation tool for a woman in the normal body mass index (BMI) range (18.5-24.9 m/kg^2^).

A website (“Me and My Baby”) was developed with advice from prenatal care providers, which required the input of a woman’s prepregnancy height and weight. The KT tool then presented a graph showing the upper and lower limits for recommended weight gain specific for each woman’s prepregnancy body mass index (BMI) according to the 2009 IOM guidelines [7] also adopted by Health Canada [16], the federal department responsible for helping Canadians maintain and improve their health, plotted on a graph against the number of weeks gestation. This graph was unique for each woman as the weekly and final optimal weight gain range was specific to her prepregnancy weight. For instance, for a woman who was 165 cm and 150 lbs, the points plotted would show two diverging lines, one from 150 lbs to 185 lbs (the upper GWG limit for a woman of her weight and BMI) and the other from 150 lbs to 175 lbs (the lower GWG limit). The tool also presented risks of excess and inadequate weight gain (care providers were encouraged to personalize them further based on the individual woman), and nutritional suggestions on how to achieve the recommended intake of 300 additional calories per day during pregnancy [17]. The website provided a print-out that could be could be folded to carry in a wallet for the participant’s own reference, while one remained in the clinical chart throughout pregnancy. This print-out was designed to facilitate prenatal discussions regarding GWG between women and their health care providers. A sample of the KT tool is shown in Figure 1.

Practitioners were encouraged to use the KT tool print-out as a starting point for GWG discussion with participants, and to write in participant-specific risks with excess or inadequate GWG that would be particularly motivating. We assessed knowledge immediately after the visit in which the tool would have been introduced to the women.

### Study population

Four local clinics in Hamilton, Ontario, a city of 520,000 [18], took part in this study: one obstetric clinic with a yearly roster of 300 pregnant patients, two midwifery practices with a combined 730 clients per year, and one family medicine practice with 50–75 pregnant patients per year. Eligibility criteria for women included being ≤ 20 weeks gestation with a live singleton pregnancy, and the ability to read English well enough to complete the surveys. Women were excluded from the study if they experienced fetal demise during their pregnancy or were pregnant with twins, triplets, or higher order multiples.

### Recruitment and study conduct

Approval from Hamilton Health Sciences/McMaster University Faculty of Health Sciences/St. Joseph’s Hospital Research Ethics Boards was obtained before the study commenced (Project #11-285). The study was explained to eligible women by the clinic staff and all participants provided written informed consent prior to study inclusion. Participants reported their prepregnancy weight and height to clinic staff who inserted this information into the KT tool website and printed two copies of the participant’s individualized tool, one for the women to bring to each appointment and one for their medical charts. Women did not log into the website themselves. Pregnant women typically had appointments with their health care providers according to the usual antenatal schedule: every month until 24 weeks gestation, every two weeks until 36 weeks gestation, and weekly thereafter until giving birth. Health care providers were instructed to plot women’s weights on the print-out of the tool at each visit and to discuss their GWG progress.

### Data collection

Self-administered surveys were conducted by participants either on paper or electronically at enrolment (≤20 weeks gestation) to evaluate knowledge outcomes and in the third trimester (approximately 36 weeks gestation) to evaluate the tool. Up to three reminders were sent for each survey, using a modified Dillman approach [19] to maximize response rates.

### Outcomes

Our primary knowledge outcome was the proportion of women who reported receiving a GWG recommendation within guidelines from their health care provider, which was assessed at the enrolment survey at ≤ 20 weeks gestation. Other GWG knowledge aspects, such as plans for weight gain and beliefs about risks with inadequate or excess GWG, were also measured at the enrolment survey ≤ 20 weeks gestation. The survey was based on a previous questionnaire conducted in the same four clinics the year before this current study [9]. Women from the previous questionnaire served as the historical control group for our analysis. We assessed participants’ evaluations of the tool, such as satisfaction with it, at approximately 36 weeks gestation.

### Historical control group

Women in the historical control group was had at least one prenatal visit, could read English sufficiently well to complete the survey, and had a live singleton gestation to be eligible to participant. Similar to the present study, the women were invited to participate in the study by clinic staff and prenatal care providers. The objectives of the study that provided the controls were to determine the proportion of women who reported being counseled at all by their health care providers and the proportion of women who were counseled appropriately according to the guidelines. The study methods and findings were published elsewhere [9].

### Sample size calculation

As our objective was to pilot the KT tool, we calculated a sample size of 130 participants as sufficient to demonstrate feasibility, based on a 15% increase in participants reporting a correct GWG recommendation from health care providers (above 12% of historical controls, n = 310) and allowing for 15% dropout as well as 15% missing data, with 85% power. KT interventions vary considerably with respect to reported absolute impact on physician behavior [20]. Grimshaw et al. [20] reported median behavior change ranging between 1% (passive educational workshops) and 21% (patient-directed interventions), thus an estimate of 15% for this intervention was appropriate as the health care providers who were involved have demonstrated above average willingness to improve knowledge and participate in shared decision-making with participants.

### Statistical analysis

Demographic variables and knowledge outcomes were compared between the KT group and the control group, including age, parity, and prepregnancy BMI using student’s *t*-test for continuous variables and chi-squared tests for categorical variables. Variables that were statistically significant in univariate logistic regression at p ≤ 0.10 were included in the multivariable logistic regression model using the enter method. In the event of high correlation (correlation coefficient ≥ 0.70) between variables, the most biologically relevant variable was selected. We maintained a relaxed rule of at least five events per variable in the initial multivariable model for each outcome [21]. The multivariable model for each outcome was based on the complete case scenario, where data from subjects with missing outcome or exposure variables were discarded. Data were analyzed using SAS 9.2 (SAS Inc, Cary, NC).

## Results

Women were recruited into the study from July to October 2011. The clinic staff kept an accurate tally of all eligible women and the number enrolled, declined and not approached for the first ten days of screening. Of the eligible women, 81% were enrolled, 17.4% declined and 1.6% were not approached. A total of 146 women were initially enrolled in the intervention study; 90% (n = 131) completed the enrolment survey and 72% (n = 105) of women completed the subsequent third trimester survey.

The women in the KT group were similar to those in the control group on most demographic factors (Table 1). The mean gestational age was 17.2 weeks (SD 5.5) at enrolment for the KT group and 30.9 weeks (SD 7.5) for the control group (P < 0.001). The mean gestational age in the present survey is much less than in the control group as it was part of our eligibility criteria for the KT group to be ≤20 completed weeks of gestation. The women in the control group did not have this requirement, as we attempted to sample consecutive women, and the visit frequency increases in later gestation, hence women were farther along in their pregnancies. Therefore, there was greater sampling of women further along their pregnancies in the control group. The groups also differed in education (secondary or less was 14.7% in the KT group and 23.5% in the control group; any post-secondary was 85.3% in KT and 76.5% in control; p = 0.039) and chronic health conditions (29.8% KT, 19.1% control; p = 0.015).

**Table 1:** **Demographic characteristics of knowledge tool group and control group**

**Variables**	**Subcategories (If applicable)**	Knowledge tool group (n = 131) N (%)*, unless otherwise indicated	**Control group (n = 310) [** 9 **]** ^**†**^ N (%)*, unless otherwise indicated	**p-value**
**Maternal age**	Mean (±SD), years	30.1 (5.5)	29.5 (5.7)	0.365
**Gestational age at enrolment**	Mean (±SD), years	17.2 (5.5)	30.9 (7.5)	<0.001^‡^
**Ethnicity**	Caucasian	96 (75.6)	229 (74.1)	0.747
**Marital status**	Married	95 (74.8)	203 (65.5)	0.156
	Common-law	17 (13.4)	53 (17.1)	
	Other	15 (11.8)	54 (17.4)	
**Education**	Secondary or less	19 (14.7)	72 (23.5)	0.039^‡^
	Any post-secondary	110 (85.3)	234 (76.5)	
**Income**	Low (< $20,000)	19 (15.7)	52 (19.8)	0.115
	Middle ($20,000 to $80,000)	40 (33.1)	106 (40.3)	
	High (> $80 000)	62 (51.2)	105 (39.9)	
**Current smoker**		14 (10.8)	32 (10.4)	0.897
**Chronic health conditions** ^**§**^		39 (29.8)	56 (19.1)	0.015^‡^
**Pregnancy history**	First time giving birth	62 (47.7)	132 (44.7)	0.437
	One previous birth	50 (38.5)	107 (36.3)	
	Two or more previous births	18 (13.9)	56 (19.0)	
**Prepregnancy BMI**	Mean (±SD), kg/m^2^	24.7 (5.7)	25.1 (6.7)	0.492
	Underweight (BMI <18.5 kg/m^2^)	11 (8.5)	20 (6.8)	0.789
	Normal weight (BMI 18.5-24.9 kg/m^2^)	67 (51.4)	164 (56.2)	
	Overweight (BMI 25.0-29.9 kg/m^2^)	33 (25.4)	65 (22.3)	
	Obese (BMI >30 kg/m^2^)	19 (14.6)	43 (14.7)	

*Abbreviations*: *BMI* Body Mass Index, *IQR* interquartile range, *N/A* not applicable, *n* sample size, *SD* standard deviation.

*Participants with missing values were discarded from percentage calculations. There may be discrepancies in percentage calculations in previously published data if missing values were not discarded.

^†^Reprinted from The American Journal of Obstetrics and Gynecology, Vol 205, Sarah D. McDonald, Eleanor Pullenayegum, Valerie H. Taylor, Olha Lutsiv, Keyna Bracken, Catherine Good, Eileen Hutton, Wendy Sword, Despite 2009 guidelines, few women report being counseled correctly about weight gain during pregnancy, Pages No. 333.e1-333.e6, Copyright (2011), with permission from Elsevier.

^‡^Statistically significant difference (p < 0.05).

^§^Included depression, anxiety, obsessive compulsive disorder, eating disorders, digestive disorders, high blood pressure, diabetes, hypoglycemia, thyroid disorders, asthma, Reynaud’s phenomenon, arthritis, pituitary microadenoma, polycystic ovary syndrome, and eczema.

### Univariate analysis on knowledge outcomes between knowledge tool and control groups

More than twice the proportion of women in the KT group reported receiving GWG counseling from their health care provider to gain a specific amount or range compared to the women in the control group (60.5% vs. 29.2%, p < 0.001) (Table 2). Eighty-six percent (85.7%) of women in the KT group compared to 47.2% in the control group reported that their health care provider discussed GWG-related topics, such as nutrition/healthy eating, appropriate weight gain, risks of gaining too much weight, and/or exercise (p < 0.001).

**Table 2:** **Univariate analysis of participant report of knowledge outcomes associated with knowledge tool group and control group**

**Variables**	**Subcategories (If applicable)**	Knowledge tool group (n = 131) N (%)*, unless otherwise indicated	**Control group (n = 310) [** 9 **]** ^**†**^ N (%)*, unless otherwise indicated	**p-value**
**Reported receiving GWG counseling from HCP**	To gain a specific amount or range^‡^	78 (60.5)	90 (29.2)	<0.001^§^
	To gain within IOM guidelines	32 (51.6)	31 (48.4)	0.722
	Discussed GWG topics with HCP**	108 (85.7)	142 (47.2)	<0.001^§^
**Believed that there were risks in gaining excess GWG**	To themselves	95 (79.2)	50 (63.3)	0.014^§^
	To their infants	75 (63.6)	42 (56.0)	0.295
**Believed that there were risks in gaining inadequate GWG**	To themselves	42 (34.4)	17 (21.3)	0.044^§^
	To their infants	73 (62.4)	28 (37.8)	0.001^§^
**Reported receiving counsel to consume an amount or range of additional calories each day by HCP**		31 (24.0)	55 (17.9)	0.139
Amount or range of additional calories recommended each day for those counseled to do so by HCP	0-100 calories	0 (0.0)	2 (3.7)	0.007^§^
	100-300 calories	23 (76.7)	23 (42.6)	
	300-500 calories	5 (16.7)	6 (11.1)	
	>500 calories	0 (0.0)	2 (3.7)	
	Could not recall how many calories	2 (6.7)	21 (38.9)	
**Reported receiving counsel to take vitamins by HCP**		12 (98.4)	301 (97.7)	0.628
**Reported discussing nutrition/healthy eating with HCP** ^**††**^		77 (61.1)	210 (69.1)	0.110
**Reported discussing appropriate weight gain with HCP** ^**††**^		82 (65.1)	142 (47.2)	0.001^§^
**Reported discussing risks of gaining too much weight with HCP** ^**††**^		38 (30.4)	79 (26.5)	0.415
**Reported discussing exercise with HCP** ^**††**^	Discussed	70 (55.6)	162 (53.8)	0.743
**Planned GWG**	Below IOM guidelines	43 (33.9)	66 (24.3)	0.124
	Within IOM guidelines	43 (33.9)	100 (36.8)	
	Above IOM guidelines	41 (32.3)	106 (39.0)	
**Difference between planned GWG and reported weight gain recommendation by HCP, kg** ^**‡‡**^	Mean of differences (SD)	0.5 (4.5)	0.9 (3.0)	0.536

*Abbreviations*: *BMI* Body Mass Index, *GWG* gestational weight gain, *HCP* health care provider, *IOM* Institute of Medicine, *IQR* interquartile range, *PDA* personal digital assistant, *SD* standard deviation.

*Participants with missing values were discarded from percentage calculations. There may be discrepancies in percentage calculations in previously published data if missing values were not discarded.

^†^Reprinted from The American Journal of Obstetrics and Gynecology, Vol 205, Sarah D. McDonald, Eleanor Pullenayegum, Valerie H. Taylor, Olha Lutsiv, Keyna Bracken, Catherine Good, Eileen Hutton, Wendy Sword, Despite 2009 guidelines, few women report being counseled correctly about weight gain during pregnancy, Pages No. 333.e1-333.e6, Copyright (2011), with permission from Elsevier.

^‡^The numerator was calculated by determining how many patients answered the question “Has your doctor, midwife, nurse practitioner or nurse made a recommendation about how much weight you should gain during pregnancy (total amount of weight)?” with either “Yes” or “I can’t remember”.

^§^Statistically significant difference (p < 0.05).

**The numerator value was calculated by counting the number of participants who responded that their health care provider discussed the following topics: nutrition/healthy eating, appropriate weight gain, risks of gaining too much weight, and exercise (the denominator is equivalent to the total number of participants who provided an answer to any of the topics mentioned).

^††^The responses to these questions are taken from the Enrolment Survey only. The questions were asked in the subsequent third trimester survey.

^‡‡^Calculated by woman's planned gestational weight gain subtracted by weight gain recommended by health care provider.

More women in the KT group than in the control group believed that there were risks to themselves with excess GWG (79.2% vs. 63.3%, p = 0.014), and risks to themselves (34.4% versus 21.3%, p = 0.044) and their infants (62.4% vs. 37.8%, p = 0.001) with inadequate GWG.

About one fourth (24.0%) of women reported being counseled to consume a specific amount or range of additional calories each day by their health care providers in the KT group, compared to 17.9% in the control group (p = 0.139).

The proportion of women who planned to gain below, within, and above the IOM guidelines in the KT group were 33.9%, 33.9%, and 32.3%, respectively, compared to the control group, 24.3%, 36.8%, and 39.0%, respectively (p = 0.124). The proportion of women who reported being recommended to gain within the IOM guidelines was 51.6% in the KT group and 48.4% in the control group (p = 0.772).

### Univariate and multivariable logistic regression on knowledge outcomes

The knowledge outcomes that were considered for the univariate and multivariable logistic regression analyses were: 1) reported receiving health care provider counseling to gain a specific amount or range of gestational weight, 2) reported discussing GWG topics with the health care provider, 3) believed that there were risks to themselves with excess GWG, 4) believed that there were risks to themselves with inadequate GWG, and 5) believed that there were risks to their infants with inadequate GWG (Tables 3, 4, 5, 6 and 7).

**Table 3:** **Univariate and multivariable analysis of variables associated with participant report of receiving health care provider counseling to gain a specific amount or range**

**Variable**	**Comparison groups**	**Crude odds ratio (95% CI)**	**P-value**	**Adjusted odds ratio (95% CI)**	**P-value**
**Study group**	KT group vs. control group [9] (reference)	3.70 (2.38-5.56)	<0.001*	3.45 (2.22-5.37)	<0.001^†^
**Maternal age**	Age as a continuous variable (per year)	1.00 (0.96-1.03)	0.865	-	-
**Ethnicity**	Caucasian vs. non-Caucasian (reference)	1.16 (0.74-1.82)	0.526	-	-
**Education**	Any post-secondary education vs. secondary education or less (reference)	1.21 (0.74-1.97)	0.440	-	-
**Income**	Low vs. middle income (reference)	1.06 (0.59-1.92)	0.845	-	-
	High vs. middle income (reference)	1.24 (0.78-1.95)	0.364	-	-
**Smoking**	Current smoker vs. non-smoker (reference)	1.19 (0.63-2.22)	0.592	-	-
**Chronic health condition**	Has chronic health condition vs. does not have chronic health condition (reference)	1.00 (0.62-1.59)	0.988	-	-
**Pregnancy history**	First birth vs. one or more previous birth (reference)	1.73 (1.17-2.57)	O0.006*	1.75 (1.15-2.67)	0.010^†^
**Prepregnancy BMI**	Underweight vs. normal weight (reference)	1.42 (0.66-3.08)	0.369	1.42 (0.64-3.17)	0.392
	Overweight vs. normal weight (reference)	1.72 (1.06-2.78)	0.028*	1.80 (1.08-3.01)	0.024^†^
	Obese vs. normal weight (reference)	1.26 (0.71-2.24)	0.434	1.32 (0.71-2.46)	0.378

*Abbreviations*: *BMI* body mass index, *CI* confidence interval, *KT* knowledge translation.

*Variable was included in multivariable logistic regression model (p ≤ 0.10).

^†^Variable was statistically significant in multivariable logistic regression model (p < 0.05).

**Table 4:** **Univariate and multivariable analysis of variables associated with participant report of discussing gestation weight gain topics with health care provider**

**Variable**	**Comparison groups**	**Crude odds ratio (95% CI)**	**P-value**	**Adjusted odds ratio (95% CI)**	**P-value**
**Study group**	KT group vs. control group [9] (reference)	6.67 (3.85-11.11)	<0.001*	7.96 (4.41-14.37)	<0.001^†^
**Maternal age**	Age as a continuous variable (per year)	0.99 (0.95-1.02)	0.467	-	-
**Ethnicity**	Caucasian vs. non-Caucasian (reference)	0.67 (0.42-1.06)	0.091*	0.52 (0.31-0.89)	0.018^†^
**Education**	Any post-secondary education vs. secondary education or less (reference)	1.52 (0.95-2.43)	0.083*	1.43 (0.83-2.47)	0.196
**Income**	Low vs. middle income (reference)	0.81 (0.45-1.43)	0.462	-	-
	High vs. middle income (reference)	1.32 (0.83-2.08)	0.239	-	-
**Smoking**	Current smoker vs. non-smoker (reference)	0.74 (0.40-1.37)	0.344	-	-
**Chronic health condition**	Has chronic health condition vs. does not have chronic health condition (reference)	1.00 (0.63-1.61)	0.987	-	-
**Pregnancy history**	First birth vs. one or more previous birth (reference)	2.24 (1.49-3.35)	0.001*	2.60 (1.66-4.07)	<0.001^†^
**Prepregnancy BMI**	Underweight vs. normal weight (reference)	1.76 (0.77-4.02)	0.178	-	-
	Overweight vs. normal weight (reference)	1.40 (0.85-2.30)	0.182	-	-
	Obese vs. normal weight (reference)	0.86 (0.49-1.53)	0.616	-	-

*Abbreviations*: *BMI* body mass index, *CI* confidence interval, *KT* knowledge translation.

*Variable was included in multivariable logistic regression model (p ≤ 0.10).

^†^Variable was statistically significant in multivariable logistic regression model (p < 0.05).

**Table 5:** **Univariate and multivariable analysis of variables associated with the belief that there are risks to themselves in gaining excess gestational weight**

**Variable**	**Comparison groups**	**Crude odds ratio (95% CI)**	**P-value**	**Adjusted odds ratio (95% CI)**	**P-value**
**Study group**	KT group vs. control group [9] (reference)	2.22 (1.16-4.17)	0.015*	1.53 (0.66-3.53)	0.323
**Maternal age**	Age as a continuous variable (per year)	1.09 (1.02-1.16)	0.008*	0.95 (0.87-1.04)	0.285
**Ethnicity**	Caucasian vs. non-Caucasian (reference)	1.15 (0.54-2.44)	0.718	-	-
**Education**	Any post-secondary education vs. secondary education or less (reference)	12.32 (5.19-29.25)	<0.001*	13.47 (4.03-44.97)	<0.001^†^
**Income**	Low vs. middle income (reference)	0.50 (0.20-1.26)	0.141	0.76 (0.25-2.30)	0.632
	High vs. middle income (reference)	4.25 (1.83-9.90)	0.001*	3.63 (1.38-9.52)	0.009^†^
**Smoking**	Current smoker vs. non-smoker (reference)	0.18 (0.07-0.47)	0.001*	0.95 (0.23-3.91)	0.947
**Chronic health condition**	Has chronic health condition vs. does not have chronic health condition (reference)	2.43 (1.01-1.16)	0.009*	2.34 (0.78-7.04)	0.132
**Pregnancy history**	First birth vs. one or more previous birth (reference)	1.65 (0.88-3.11)	0.122	-	-
**Prepregnancy BMI**	Underweight vs. normal weight (reference)	0.48 (0.17-1.39)	0.175	-	-
	Overweight vs. normal weight (reference)	0.92 (0.42-1.98)	0.825	-	-
	Obese vs. normal weight (reference)	0.58 (0.24-1.43)	0.238	-	-

*Abbreviations*: *BMI* body mass index, *CI* confidence interval, *KT* knowledge translation.

*Variable was included in multivariable logistic regression model (p ≤ 0.10).

^†^Variable was statistically significant in multivariable logistic regression model (p < 0.05).

**Table 6:** **Univariate and multivariable analysis of variables associated with the belief that there are risks to themselves in gaining inadequate gestational weight**

**Variable**	**Comparison groups**	**Crude odds ratio (95% CI)**	**P-value**	**Adjusted odds ratio (95% CI)**	**P-value**
**Study group**	KT group vs. control group [9] (reference)	1.96 (1.01-3.70)	0.046*	1.99 (0.92-4.30)	0.080
**Maternal age**	Age as a continuous variable (per year)	1.00 (0.94-1.06)	0.991	-	-
**Ethnicity**	Caucasian vs. non-Caucasian (reference)	0.78 (0.38-1.64)	0.522	-	-
**Education**	Any post-secondary education vs. secondary education or less (reference)	2.11 (0.82-5.42)	0.121	-	-
**Income**	Low vs. middle income (reference)	1.07 (0.36-3.19)	0.910	1.06 (0.33-3.40)	0.916
	High vs. middle income (reference)	2.18 (1.01-4.70)	0.046*	2.18 (0.97-4.93)	0.061
**Smoking**	Current smoker vs. non-smoker (reference)	0.54 (0.17-1.68)	0.286	-	-
**Chronic health condition**	Has chronic health condition vs. does not have chronic health condition (reference)	1.06 (0.52-2.17)	0.874	-	-
**Pregnancy history**	First birth vs. one or more previous birth (reference)	1.46 (0.79-2.70)	0.229	-	-
**Prepregnancy BMI**	Underweight vs. normal weight (reference)	0.27 (0.07-0.99)	0.048*	0.34 (0.09-1.30)	0.115
	Overweight vs. normal weight (reference)	0.44 (0.21-0.90)	0.025*	0.44 (0.19-0.98)	0.044^†^
	Obese vs. normal weight (reference)	0.05 (0.01-0.37)	0.003*	0.05 (0.01-0.36)	0.003^†^

*Abbreviations*: *BMI* body mass index, *CI* confidence interval, *KT* knowledge translation.

*Variable was included in multivariable logistic regression model (p ≤ 0.10).

^†^Variable was statistically significant in multivariable logistic regression model (p < 0.05).

**Table 7:** **Univariate and multivariable analysis of variables associated with the belief that there are risks to their infants in gaining inadequate gestational weight**

**Variable**	**Comparison groups**	**Crude odds ratio (95% CI)**	**P-value**	**Adjusted odds ratio (95% CI)**	**P-value**
**Study group**	KT group vs. control group [9] (reference)	2.70 (1.49-5.00)	0.001*	2.48 (1.14-5.37)	0.021^†^
**Maternal age**	Age as a continuous variable (per year)	0.98 (0.93-1.04)	0.565	-	-
**Ethnicity**	Caucasian vs. non-Caucasian (reference)	1.82 (0.89-3.70)	0.105	-	-
**Education**	Any post-secondary education vs. secondary education or less (reference)	4.43 (1.79-10.97)	0.001*	5.36 (1.50-19.17)	0.010^†^
**Income**	Low vs. middle income (reference)	0.53 (0.20-1.40)	0.199	0.56 (0.17-1.88)	0.350
	High vs. middle income (reference)	1.96 (1.00-3.85)	0.050*	1.52 (0.67-3.45)	0.315
**Smoking**	Current smoker vs. non-smoker (reference)	0.34 (0.11-1.00)	0.051*	0.87 (0.18-4.15)	0.866
**Chronic health condition**	Has chronic health condition vs. does not have chronic health condition (reference)	3.02 (1.44-6.32)	0.003*	3.39 (1.32-8.69)	0.011^†^
**Pregnancy history**	First birth vs. one or more previous birth (reference)	2.43 (1.36-4.36)	0.003*	2.10 (1.00-4.42)	0.051
**Prepregnancy BMI group**	Underweight vs. normal weight (reference)	0.50 (0.17-1.46)	0.206*	0.82 (0.21-3.14)	0.775
	Overweight vs. normal weight (reference)	0.45 (0.23-0.89)	0.022*	0.45 (0.20-1.05)	0.064
	Obese vs. normal weight (reference)	0.14 (0.05-0.37)	<0.001*	0.09 (0.03-0.31)	<0.001^†^

*Abbreviations*: *BMI* body mass index, *CI* confidence interval, *KT* knowledge translation.

*Variable was included in multivariable logistic regression model (p ≤ 0.10).

^†^Variable was statistically significant in multivariable logistic regression model (p < 0.05).

Biologically plausible variables selected for both univariate and multivariable analyses for each knowledge outcome included: study group (intervention vs. control group), maternal age (continuous variable, per year), ethnicity (Caucasian vs. non-Caucasian), education (any post-secondary education vs. secondary education or less), income (middle income as reference group), chronic health condition (yes vs. no), pregnancy history (first birth vs. one or more previous births) and prepregnancy BMI (normal weight as reference group).

In multivariable analyses for knowledge outcomes, women who reported receiving health care provider counseling to gain a specific amount or range were more likely to be in the KT group (adjusted odds ratio [AOR] 3.45, 95% confidence interval [CI] 2.22-5.37), to be giving birth for the first time (AOR 1.75, 95% CI 1.15-2.67), and to be overweight (AOR 1.80, 95% CI 1.08-3.01). Women who reported discussing GWG topics with their health care providers were more likely to be in the KT group (AOR 7.96, 95% CI 4.41-14.37) and to be giving birth for the first time (AOR 2.60, 95% CI 1.66-4.07), and were less likely to be Caucasian (AOR 0.52, 95% CI 0.31-0.89). However, women who believed that there were risks to themselves with excess GWG differed in education (AOR 13.47, 95% CI 4.03-44.97 for any post-secondary education) and income (AOR 3.63, 95% CI 1.38-9.52 for high income), but not by study group. Additionally, women who believed that there were risks to themselves with inadequate GWG only differed in BMI with normal weight women as the reference group (AOR 0.44, 95% CI 0.19-0.98 for overweight; and AOR 0.05, 95% CI 0.01-0.36 for obese women). Women who believed that there were risks to their infants with inadequate GWG were more likely to: be in the KT group (AOR 2.48, 95% CI 1.14-5.37), have any post-secondary education (AOR 5.36, 95% CI 1.50-19.17), have a chronic health condition (AOR 3.39, 95% CI 1.32-8.69), and be normal weight than obese (AOR 0.09, 95% CI 0.03-0.31).

With regard to the missing data, of the outcomes considered for multivariable analysis, there were no significant differences in percent missing between the KT group and control group for “reported receiving health care provider counseling to gain a specific amount or range” (1.5% KT and 0.7% control), reported discussing gestational weight gain topics with health care provider” (3.8% KT and 2.9% control), and “believed that there are risks to themselves in gaining excess gestational weight gain” (8.4% KT and 15.6% control). There were differences between the outcomes of “believed that there are risks to themselves in gaining inadequate gestational weight” (6.9% KT and 15.6% control) and “believed that there are risks to their infants in gaining gestational weight” (10.7% and 21.1%). The respondents in both groups were more likely to live in high-income than middle- or low-income households than those who did not respond for these outcomes (respectively: for “risks to themselves”, 94.2% vs. 82.7%, p = 0.01; and for “risks to their infants”, 90.8% vs. 77.3%, p = 0.008).

### Knowledge translation tool evaluation

Women’s evaluation of the KT tool is shown in Table 8. About half of women reported “definitely yes” or “probably yes” to recommending the tool to a friend (49.5%). Most women felt the tool could be improved by one or more changes, including having a bigger graph (54.3%), more information on food servings (43.8%), or more information on exercise (30.5%), while 14.3% of women felt the tool should remain unchanged.

**Table 8:** **Participant’s evaluation of knowledge translation tool**

**Variables**	**Subcategories (if applicable)**	N (%)*, unless otherwise indicated
**Would recommend the KT tool to a friend**	Definitely not	0 (0.0)
	Probably not	25 (25.3)
	Don’t know	25 (25.3)
	Probably yes	42 (42.4)
	Definitely yes	7 (7.1)
**Suggested improvements to KT tool**	Bigger graph	57 (54.3)
	More information on food servings	46 (43.8)
	More information on exercise	32 (30.5)
	Other	15 (14.3)
	Should remain unchanged	15 (14.3)
**HCP showed KT tool**	Never	35 (35.4)
	Occasionally	18 (18.2)
	About half the time	9 (9.1)
	Usually	13 (13.1)
	Always	24 (24.2)
**Marker of KT tool**	Participant (pregnant woman)	40 (40.4)
	Nurse/Nurse Practitioner	15 (15.2)
	Doctor	11 (11.1)
	Midwife	23 (23.2)
	Other	10 (10.1)
**Preference if using knowledge KT in the future**	Only mark weight on the tool themselves	11 (11.1)
	Only have HCP mark weight on the tool	28 (28.3)
	Have HCP mark their weight on the tool in their chart, and mark their weight on their own copy of the tool themselves	60 (60.6)
**Preference in marking weight**	On their own paper copy of tool	39 (54.9)
	On an “app” (application) for their hand-held electronic device	22 (31.0)
	Online on a website on a computer	9 (12.7)
	Other	1 (1.4)
**Were given a copy of KT tool**	No	16 (16.2)
	Yes	83 (83.8)
**Checked their weight gain on KT tool**	About once a week	4 (4.8)
	About every two weeks	9 (10.8)
	About once per month	17 (20.5)
	Before (or at) every clinic visit	41 (49.4)
	Other	12 (14.5)

*Abbreviations*: *HCP* health care provider, *KT* knowledge translation, *n* sample size.

*Participants with missing values were discarded from percentage calculations.

When asked if their health care provider showed them the tool at each visit, 24.2% of women reported “always”, 13.1% “usually”, 9.1% “about half the time”, 18.2% “occasionally” and 35.4% “never”.

Sixty percent (59.6%) of women reported that their health care providers marked their weights on the tool, and 40.4% of women reported that they marked their own weights. If using the tool in the future, most women (60.6%) preferred to have both their health care provider mark their weights in their chart and also mark their weight themselves on their own copy. The majority of women would prefer to mark their weights on their own paper copy (54.9%) or on an “app” for their hand-held electronic device (31.0%) if they were to use the tool in the future.

Most women (83.8%) reported being given their own copy of the tool, and checked their weight gain on the tool about once a week (4.8%), every two weeks (10.8%), once per month (20.5%), at or before every clinic visit (49.4%).

## Discussion

Women in the KT group had better GWG knowledge outcomes compared to women in the control group, even after adjusting for potential confounders. More women in the KT group reported receiving health care provider counseling to gain a specific amount or range and discussing GWG topics with their health care providers, and correctly believed that there were risks to their infants with inadequate GWG. We demonstrated that a simple, sustainable, easily transferable tool improves pregnant women’s knowledge of appropriate GWG. However, a substantial proportion of women still did not report receiving any recommendation from the care provider, did not have knowledge about risks with inappropriate GWG, and had not been shown the tool.

A previous study that also measured knowledge outcomes showed that knowledge-based interventions were effective. In a 2012 study, Wilkinson et al. [22] conducted a 60-minute presentation to the intervention group, which included a component of goal-setting and self-monitoring with respect to dietary and physical activity behaviors during pregnancy, and found that the percentage of women with GWG goals within guidelines was significantly greater in the intervention group (8% difference, p = 0.009). A systematic review by Brown et al. in 2012 of five randomized trials used goal-setting for GWG alongside modification to diet and/or physical activity [23]. The authors concluded that planning weight gain in conjunction with changes in eating or exercise habits may be effective in lowering excess GWG. Interventions with planned weight gain goals, such as with our study, may be more effective than lifestyle changes alone.

Forty-four percent of women reported that the KT tool could be improved by having more information on food servings. Tanentsapf and colleagues also suggested in a systematic review that interventions that included face-to-face nutritional counseling and recommendations on caloric intake may be more successful at reducing total gestational weight gain than those that do not include these components, and education on appropriate weight gain in early pregnancy may increase the rate of compliancy [24].

More women in the KT group correctly believed that there were risks to themselves with excess GWG, and risks to themselves and their infants with inadequate GWG in the univariate analysis. In contrast, there was no difference between the KT group and control group in the belief that there were risks to their infants with excess GWG, possibly due to the KT tool not specifying risks factors for infants related to excess weight gain. However, more women in the KT group believed that there were risks to themselves with inadequate GWG despite the tool not stating such risks (Figure 1). Most of the literature suggests there is no increased risk for women who gain below the guidelines, although a study suggests that there may be increased risk of maternal fever, active-phase arrest and the need for episiotomy [25].

Despite the use of the KT tool, only half of women reported that their health care provider counselled them to gain within the IOM guidelines. It is possible that participants are not correctly recalling the amount of weight recommended by their provider [26]. In addition, women may weigh the advice of family or friends, or influences of their culture more heavily, which could be discordant to health care provider recommendations [27,28]. This could explain why 52% of women reported receiving health care provider counselling to gain within the guidelines, but 34% actually planned to gain within. Another explanation is the sensitivity of the topic of GWG. Health care providers may be concerned that women may be offended, angered, saddened or embarrassed when discussing GWG [27], however in previous work, we noted that 84% of women reported being ‘comfortable’ or ‘very comfortable’ discussing weight related issues with their care provider [9]. They may also be uncertain of the effectiveness of their counselling [27,28]. These factors coupled with time constraints of the prenatal appointment [10] may cause limited counselling on gaining within the guidelines. To note, over one third of the participants reported that their health care provider “never” showed them the KT tool. A way to address some of these challenges could potentially lie in focusing on the baby’s health during counselling. Pregnant women may be more determined to make positive lifestyle changes over the concern of their baby [29]. Change could be easier the greater the perceived risk of the infant [29]. Better awareness the infant risks of excess GWG could make the weight gain recommendations more memorable and impactful to the women, and could address issues of sensitivity, as the focus is away from the women’s weight but rather optimizing outcomes for the infant.

We have some limited data from the survey of health care providers, in which 34.8% responded that they or their office staff would refer to the KT graph at about half of the patient’s visits, and 30.4% responded ‘almost never’. About one quarter of care providers responded that they almost never referred to the graph because it took too much time, while a similar proportion stated because the patient was not interested, and the remainder stated it was due to other issues such as the graph being too small, they had referred the patient to a dietician.

The majority (64.6%) of women reported that their health care provider had shown them the KT tool, which presented the range of the recommended GWG and gave the health care provider an opportunity to discuss it. Another study in a similarly predominantly white, middle-class population showed that women who did not receive advice on weight gain were more likely to gain less (AOR 1.8, 95% CI 1.3-2.5) or more (AOR 2.0, 95% CI 1.5-2.7) than recommended, compared to women who were advised according to the recommendations [30]. Women who gained gestational weight above the IOM guidelines had greater than three times the odds of having received a recommendation to gain above [30] and women who gained weight below the guidelines had greater than three times the odds of having received recommendations to gain below [30]. Brawarsky et al. observed that 71% of women who received recommendations above guidelines gained weight above the guidelines [31]. Herring et al. reported that in a sample composed of women of low-socioeconomic status, those who gained weight above guidelines had almost six times the odds of having received a recommendation that was either above or below instead of within guidelines [32].

Strengths of our study include the longitudinal nature of the study, as we followed the same women throughout their pregnancies to use their evaluation of the KT tool at the end of pregnancy. Although we used historical controls from the year before, they were from the same prenatal clinics and had the same health care providers. Physicians and midwives involved in the development of the tool were knowledge users, and therefore had an understanding of how to utilize the tool in their prenatal care appointments.

Limitations of our study include the relatively small sample size, although it was powered for our primary outcome. The use of historical controls also presented differences between the KT and the control groups, including gestational age, education level, and chronic health conditions. The gestational age at recruitment of the intervention group was earlier than the control group due to the eligibility criteria, and thus could lead to recall bias and bias the results in favor of the intervention group. However it might have also led to bias in the opposite direction, given that women in the control group had had many more opportunities to have discussion about GWG with their care providers. Knowledge was assessed in the KT tool group at a mean of 17 weeks just after recruitment, and in the control group at a mean of 31 weeks. The women in the KT tool group would have typically had 2 regularly scheduled visits before being recruited, approximately four weeks apart (e.g. 12 and 16 weeks). At 32 weeks, the standard visit schedule includes 8 visits.

It is possible that the publication of the results of the historical control survey may have resulted in clinical practice change, so that the difference in knowledge that we found may have been due to factors other than the intervention. However, given that the publication of clinical practice guidelines has been proven to be relatively ineffective at changing practice [33], it is not clear that the publication of a single paper would have done so.

Another potential limitation is that prepregnancy weight was self-reported. However, this is what is done in clinical settings, in which the patients provide their prepregnancy weight. Also, this occurred in *both* the intervention and the control groups. Hence whatever bias might occur had the potential to do so equally in both groups.

We had some missing data, which was addressed through complete case analysis, but there were differences in income in those who responded compared to those who did not respond. Our study population was limited to English-speaking women with singletons and the results are generalizable only to this group. In addition, the majority of women were well-educated and of higher socioeconomic status.

## Conclusion

Pregnant women who received a simple knowledge translation tool reported higher rates of counseling on GWG and were more aware of the risks of gaining outside the appropriate range. This pilot study demonstrated that knowledge interventions for GWG may be effective. Further research is needed on the association between GWG education and GWG.

## Abbreviations

AOR: Adjusted odds ratio
BMI: Body mass index
CI: Confidence interval
GWG: Gestational weight gain
HCP: Health care provider
IOM: Institute of medicine
SD: Standard deviation
